# Cerebellar tDCS Does Not Enhance Performance in an Implicit Categorization Learning Task

**DOI:** 10.3389/fpsyg.2017.00476

**Published:** 2017-04-05

**Authors:** Marie C. Verhage, Eric O. Avila, Maarten A. Frens, Opher Donchin, Jos N. van der Geest

**Affiliations:** ^1^Department of Neuroscience, Erasmus MCRotterdam, Netherlands; ^2^Erasmus University CollegeRotterdam, Netherlands; ^3^Department of Biomedical Engineering, Ben-Gurion University of the NegevBe’er Sheva, Israel

**Keywords:** brain stimulation, cerebellum, cognition, information integration, humans

## Abstract

**Background:** Transcranial Direct Current Stimulation (tDCS) is a form of non-invasive electrical stimulation that changes neuronal excitability in a polarity and site-specific manner. In cognitive tasks related to prefrontal and cerebellar learning, cortical tDCS arguably facilitates learning, but the few studies investigating cerebellar tDCS, however, are inconsistent.

**Objective:** We investigate the effect of cerebellar tDCS on performance of an implicit categorization learning task.

**Methods:** Forty participants performed a computerized version of an implicit categorization learning task where squares had to be sorted into two categories, according to an unknown but fixed rule that integrated both the size and luminance of the square. Participants did one round of categorization to familiarize themselves with the task and to provide a baseline of performance. After that, 20 participants received anodal tDCS (20 min, 1.5 mA) over the right cerebellum, and 19 participants received sham stimulation and simultaneously started a second session of the categorization task using a new rule.

**Results:** As expected, subjects performed better in the second session than in the first, baseline session, showing increased accuracy scores and reduced reaction times. Over trials, participants learned the categorization rule, improving their accuracy and reaction times. However, we observed no effect of anodal tDCS stimulation on overall performance or on learning, compared to sham stimulation.

**Conclusion:** These results suggest that cerebellar tDCS does not modulate performance and learning on an implicit categorization task.

## Introduction

Over the past decades, transcranial Direct Current Stimulation (tDCS) has shown to be a promising tool for enhancing motor and cognitive learning in humans (Jacobson et al., 2012; Ferrucci and Priori, 2014). While this has been shown for both cerebellar and supratentorial cortical tDCS in motor tasks, there have been inconsistent reports of enhanced cognitive learning following cerebellar stimulation.

Those studies that have examined cerebellar tDCS in cognitive tasks are focussed on explicit learning tasks, and this may be a partial explanation for the conflicting results. Explicit learning is a conscious process that involves predominantly the prefrontal cortex (Ashby and Maddox, 2005). Implicit learning, on the other hand, is a subconscious process in which the cerebellum is more substantially involved (Ito, 2008). With this dissociation in mind, we explored the cerebellar role in cognition by applying tDCS to the cerebellum in an implicit version of a learning task.

Transcranial Direct Current Stimulation over the cortex has shown different effects on cognitive learning. Compared to cathodal tDCS, the facilitating effect of anodal stimulation is more established (Jacobson et al., 2012). Therefore, most brain stimulation research has investigated the effect of anodal tDCS over the prefrontal cortex and found facilitating effects on explicit problem solving, working memory, and language tasks (Monti et al., 2013; Brunoni and Vanderhasselt, 2014; Coffman et al., 2014). Also, several studies have investigated the effect of anodal tDCS on categorization; however, results are inconsistent. Stimulation over the left inferior frontal cortex improved performance on a simple, explicit categorization task (Lupyan et al., 2012). However, anodal (and cathodal) tDCS over the dorsolateral prefrontal cortex impaired categorization performance in a prototype distortion task (Ambrus et al., 2011). Conflicting results were also found on a probabilistic classification task. A study found facilitating effects with anodal tDCS over the prefrontal cortex, (Kincses et al., 2004). However, a recent study was not able to reproduce this effect, highlighting the importance of replication (Seyed Majidi et al., 2017).

The prefrontal cortex is primarily involved in cognitive processes; however, the role of the cerebellum in cognition is currently under debate. Imaging studies have consistently shown cerebellar activation in various cognitive tasks (Nitsche et al., 2003; Blackwood et al., 2004; Hayter et al., 2007; Tomasi et al., 2007; Helie et al., 2010; Stoodley et al., 2012; Balsters et al., 2013; Lam et al., 2013; Davis et al., 2014; E et al., 2014) including categorization tasks (Patalano et al., 2001; Milton et al., 2009). On the other hand, inconsistent results have been found in patients with cognitive impairment due to cerebellar lesions. Lesion studies have shown impaired categorization capabilities (Bolcekova et al., 2012) and abstract reasoning skills (Schmahmann and Sherman, 1998) in cerebellar patients, whereas other studies have not found any differences between lesion patients and healthy controls (Maddox et al., 2005; Ell and Ivry, 2008). These findings are in line with the notion of the cerebellum as an automating system. The prefrontal, or motor, cortex is essential for learning and the cerebellum automates these cognitive, or motor, processes; damage to the cerebellum results in impaired skilled performance and automaticity (Ramnani, 2006; Balsters and Ramnani, 2008). Results of lesion studies should nonetheless be interpreted with care (Timmann and Daum, 2010), especially since cause and location of cerebellar lesions vary widely between patients (Gottwald et al., 2004).

This raises the question what the effect is of cerebellar tDCS on cognitive learning. A handful studies investigated the effect of cerebellar tDCS on explicit cognitive learning and reported promising effects. tDCS over the cerebellum impaired reaction time in a working memory task (Ferrucci et al., 2008) and impaired performance in a verbal working memory task (Boehringer et al., 2013). Another study found facilitation on verbal responses in a verb generation task and addition task with cathodal tDCS (Pope and Miall, 2012). The authors concluded that direct current stimulation over the right cerebellum affects working memory and attention differently depending on task difficulty and suggested that the cerebellum is capable of releasing cognitive resources when tasks become demanding. However, a recent cerebellar tDCS study investigating cognitive load in a working memory task was unable to confirm this hypothesis (van Wessel et al., 2016). Moreover, a small sample–sized study investigating implicit cognitive learning in a probabilistic weather prediction task was unable to alter performance with cerebellar tDCS (Seyed Majidi et al., 2017). The effect of cerebellar tDCS on various cognitive tasks have shown conflicting results. The majority of the aforementioned studies investigated the effects of cerebellar tDCS in explicit learning tasks. However, we believe results will be more consistent in an implicit learning task due to the substantial involvement of the cerebellum in implicit learning (Ito, 2008). Moreover, previous research has shown modulatory effects of cerebellar tDCS on implicit learning in motor tasks (Galea et al., 2011; Ferrucci et al., 2013).

We conducted a pilot study investigating anodal, cathodal, and sham stimulation over the prefrontal cortex and cerebellum in a rule-based (explicit) and information-integration (implicit) categorization task. The pilot results in our implicit categorization task showed improved accuracy scores in the cerebellar groups for anodal stimulation compared to cathodal and sham stimulation (Verhage et al., 2014). The pilot study has a small sample size, and results should, therefore, be interpreted with care (Slavin and Smith, 2009). We decided to partly replicate this pilot study in a larger sample. In this present study, based on the established effect of anodal tDCS (Jacobson et al., 2012) and our previous results (Verhage et al., 2014), we investigate the effects of tDCS (anodal and sham) over the cerebellum in an information-integration categorization task. We expect that anodal cerebellar tDCS will enhance performance during an implicit categorization task.

## Materials and Methods

### Subjects

Forty-one healthy right-handed subjects were recruited to participate in a single stimulation condition (age range: 20–31 years). The number of subjects was calculated according to the effect size observed in our pilot study (Cohen’s *d* = 0.56, alpha 5%, power 80%, yielding >19 subjects per group). All subjects were college students and naïve to the experiment. Subjects were right handed, had no history of neurological deficits, no metal plate implanted in or near the head, and no history of chronic drug abuse. In return for their participation, subjects received course credit, and the highest scoring subjects received a small financial reward.

### Experimental Design

**FIGURE 1:**
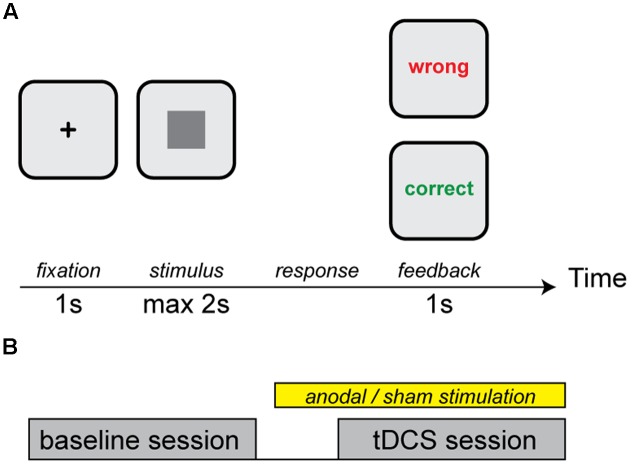
**(A)** An example of a trial. Each trial started with a fixation cross followed by a square of a specific luminance and size. Participants received feedback on their categorization choice. **(B)** Design of the experiment. Subjects started with a baseline session followed by the transcranial Direct Current Stimulation (tDCS) session during which participants received either anodal cerebellar or sham tDCS.

At arrival, participants signed the informed consent form and were seated before a laptop. The categorization task was explained by the experimenter and again on the computer screen. To determine basic categorization performance, subjects started with a baseline measurement without stimulation. After that, subjects executed an additional categorization task with tDCS (**Figure 1**). After every categorization task, subjects were asked to briefly describe how they categorized the gray squares, to see if they indeed used both dimensions (see task below) rather than one.

Subjects received anodal or sham stimulation over the right cerebellum. The study design had a randomized, single-blind, sham-controlled, between-subjects design. The entire experiment took approximately 1 h.

### Categorization Task

The categorization task was performed on a 15-inch laptop computer. Stimulus presentation was done by custom-made software written in MATLAB (MathWorks, Natick, MA, USA). The stimuli consisted of a square presented on a white background. Squares varied on two dimensions: size and luminance. A square could have one out of ten different sizes (side length ranged from 55 to 119 pixels) and one out of ten different luminances (black to nearly white), making up 100 different squares.

In a single trial, a fixation point was shown on the center of the screen, followed by a square. The participant had to assign the square to category A by pressing the “Z” key on the keyboard, or to category B by pressing the “M” key, within 2 s. After pressing a key, the subject received feedback for 1 s (**Figure 1B**). A short break was introduced every 50 trials. In total, 300 trials were presented in each stimulation condition (100 squares with three repetitions).

Subjects learned to categorize the squares without prior knowledge of how the categories were divided. During categorization, subjects aimed to employ an internal rule to classify the stimuli. Therefore, a large amount of simple, confusable stimuli were used to prevent subjects from remembering individual examples (Ashby and Ell, 2001; Rouder and Ratcliff, 2006). The task used was an implicit category learning task; the goal is to combine information from two or more stimulus characteristics (information integration) to maximize accuracy, where the optimal rule is difficult or impossible to describe verbally (Ashby and Maddox, 2005).

**FIGURE 2:**
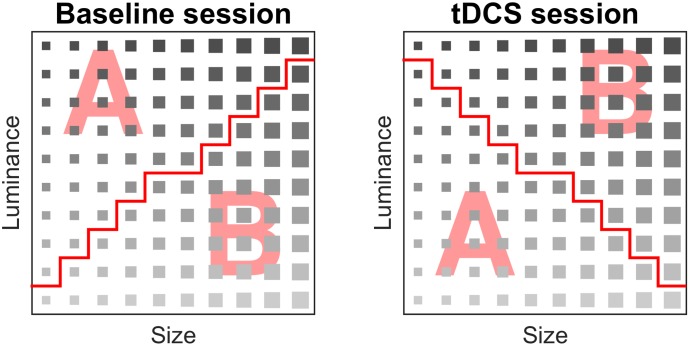
**Stimulus matrix of the baseline and tDCS session**. A stimulus was assigned to category A or B. The red line denotes separation between categories A and B, i.e., the rule that participants implicitly learned during the task.

The rule dividing the categories was a combination of two stimuli dimensions (luminance and size). The categories were linearly separable. Subjects performed two implicit categorization tasks of the same complexity level; a baseline measurement without stimulation and an additional measurement with stimulation. In every categorization task, the same stimuli were used; however, the rule that divided the categories was different (**Figure 2**).

### Transcranial Direct Current Stimulation (tDCS)

Transcranial Direct Current Stimulation (tDCS) was administered with a CE-certified constant current stimulator (neuroConn, Ilmenau, Germany) through two annular sintered Ag/AgCl 12-mm diameter electrodes (MedCaT, Erica, The Netherlands) with highly conductive gel (Signa Gel; Parker Laboratories, Fairfield, NJ, USA). The target electrode was placed over the right cerebellum 3 cm lateral to the inion, and reference electrode was placed over the ipsilateral buccinator muscle. The tDCS was applied for 20 min with 1.5 mA (current density of 1.33 mA/cm^2^). Sham stimulation was ramped up to 1.5 mA for 30 s and turned down after 60 s.

### Data Analysis

Participants who did not show a clear categorization strategy were removed from analysis. Data were analyzed with SPSS (v20.0, IBM Corp., Armonk, NY, USA) and MATLAB (MathWorks, Natick, MA, USA). Accuracy scores and reaction time were measured as dependent variables. For each session, we calculated the percentage of correct responses for each block of 25 trials (12 in total). We also calculated mean reaction time for each block. In addition, we calculated the percentage of correct responses, mean response times, and also the variance of the reaction times for the whole baseline session and for the whole stimulation session. Baseline performance was determined to assess potential group differences.

### Statistical Approach

The overall effect of tDCS was assessed by a mixed ANOVA with one between-participant factor stimulation condition (two levels: anodal and sham) and one within-participant factor session (two levels: baseline and tDCS). *T*-tests were used to investigate baseline performance and follow-up comparisons. Additional analyses were performed comparing the first and last block. Analyses were performed for accuracy scores and response times separately. Performance was based on the average accuracy, reaction time, and reaction time variance for every subject.

The effect of tDCS on learning was assessed by a mixed ANOVA with one between-participant factor stimulation condition (two levels: anodal and sham) and one within-participant factor block (12 levels: 12 blocks of 25 trials). In case of sphericity violations, we report corrected estimations of the degrees of freedom. All reported values are means ± standard deviations. The level of significance was set at α = 0.05.

In addition to the frequentist method using Null Hypothesis Significance Testing (NHST), we also performed a Bayesian analysis to investigate tDCS effects on accuracy and reaction time (similar to Smittenaar et al., 2014). Inferences from Bayesian analyses are more informative than NHST, especially in the absence of experimental effects (Kruschke, 2014). Here, we used it to statistically assess the observed data with a Bayesian model comparison analysis by fitting three models to our data: a null model, a main model, and an extended model. The null model incorporates parameter block (to assess overall learning), the main model additionally incorporates the parameter stimulation condition (to assess the overall effect of tDCS), and the extended model further incorporates the interaction parameter block-by-stimulation condition (to assess the effect of tDCS on learning). To investigate our hypothesis that anodal cerebellar tDCS enhances implicit categorization learning task, we compared the null model to the main model, assessing overall effects of tDCS, and we compared the main model to the extended model, assessing an effect of tDCS on learning (similar to Smittenaar et al., 2014).

## Results

Two subjects were removed from analysis because they did not show a clear categorization strategy based on two dimensions, leaving 39 subjects for analysis (13 males, 28 females; mean age ± SD: 22.8 ± 2.3 years, age range: 20–31 years). Twenty participants formed the anodal tDCS group, and 19 participants belonged to the sham tDCS group.

### Accuracy

Before stimulation, participants in both groups had similar accuracy scores (sham = 0.75 ± 0.10%, anodal = 0.77 ± 0.08%) in the baseline measurement [*t* (37) = 0.50, *p* = 0.62, *d* = 0.22]. The ANOVA showed a significant effect of session [*F*(1,37) = 10.50, *p* < 0.005, η^2^ = 0.22]. On average, participants performed better in the tDCS session compared to the baseline measurement (baseline = 0.74 ± 0.05%, tDCS = 0.77 ± 0.05%). The main effect of stimulation condition on accuracy was not significant [*F*(1,37) = 2.11, *p* = 0.16, η^2^ = 0.06]. Moreover, the interaction effect between stimulation condition and session was not significant [*F*(1,37) = 1.05, *p* = 0.31, η^2^ = 0.03].

**FIGURE 3:**
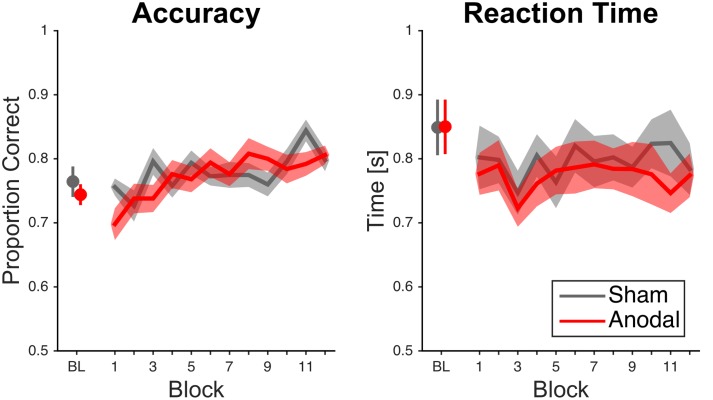
**Raw mean accuracy scores and reaction times over time for cerebellar tDCS**. Data analysis on accuracy scores showed an interaction effect for block and stimulation condition; however, significance did not survive Bonferroni correction. Error bars and error areas denote standard error of mean (BL = last block of baseline).

Subjects performed better over time (**Figure 3**). The ANOVA showed a significant effect of block [*F*(11,407) = 4.33, *p* < 0.001, $\eta_{p}^{2}$ = 0.11]. This was supported by the fact that participants performed better in the last block (0.80 ± 0.07%) than in the first block (0.73 ± 0.09%). The main effect of stimulation condition was not significant [*F*(1,37) = 0.16, *p* = 0.69, $\eta_{p}^{2}$ < 0.01]. The interaction between block and stimulation condition was significant [*F*(11,407) = 1.95, *p* = 0.03, $\eta_{p}^{2}$ = 0.05], but *post hoc* comparisons per block showed no significant differences between sham and anodal stimulation.

### Reaction Time

Before stimulation, participants had similar reaction times (sham = 0.87 ± 0.14 s, anodal = 0.88 ± 0.12 s) in the baseline measurement [*t* (37) = 0.23, *p* = 0.82, *d* = 0.15]. The ANOVA showed a significant effect of session [*F*(1,37) = 29.12, *p* < 0.001, η^2^ = 0.44]. On average, participants performed faster in the tDCS session (baseline = 0.87 ± 0.13 s, tDCS = 0.78 ± 0.13 s). The main effect of stimulation condition was not significant [*F*(1,37) = 0.03, *p* = 0.86, η^2^ < 0.01]. Moreover, the interaction effect between stimulation condition and session was not significant [*F*(1,37) = 1.04, *p* = 0.32, η^2^ = 0.03].

The two groups had similar reaction time variance (both groups: 0.03 ± 0.02 s) in the baseline measurement [*t* (37) = 0.74, *p* = 0.46]. The ANOVA showed a significant effect for session [*F*(1,37) = 14.14, *p* < 0.005, η^2^ = 0.28]. Subjects’ responses were less variable in the tDCS session compared to the baseline (baseline = 0.03 ± 0.02 s, tDCS = 0.01 ± 0.01 s). The main effect for stimulation condition was not significant [*F*(1,37) = 0.39, *p* = 0.54, η^2^ = 0.01]. Moreover, the interaction effect between stimulation condition and session was not significant [*F*(1,37) = 0.64, *p* = 0.43, η^2^ = 0.02].

Subjects did not respond faster over time as the ANOVA did not show a significant effect of block [*F*(7.34,271.46) = 0.91, *p* = 0.50, $\eta_{p}^{2}$ = 0.02; Greenhouse–Geisser correction, ε = 0.67; **Figure 3**]. This was further supported by the fact that participants did not perform faster in the last block (0.78 ± 0.16 s) than in the first block [0.79 ± 0.18 s; *t* (38) = 0.32, *p* = 0.75, *d* = 0.06]. The main effect of stimulation condition was not significant [*F*(1,37) = 0.32, *p* = 0.57, $\eta_{p}^{2}$ < 0.01]. Moreover, the interaction between block and stimulation condition was not significant [*F*(7.34,271.46) = 0.46, *p* = 0.87, $\eta_{p}^{2}$ = 0.01; Greenhouse–Geisser correction, ε = 0.67].

**FIGURE 4:**
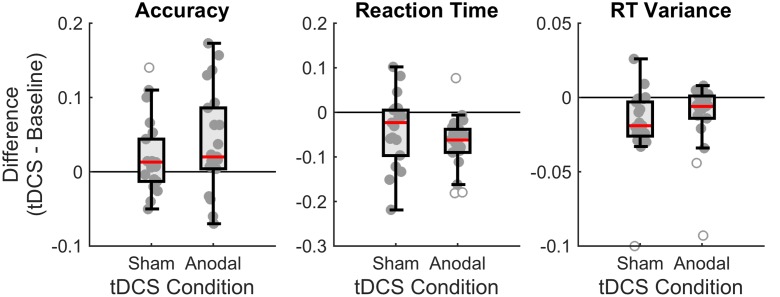
**Boxplots of the differences between baseline and tDCS session for overall accuracy, reaction time (RT), and RT variance, for the two stimulation conditions (sham and anodal)**. Each dot denotes an individual subject. The boxplot indicates the median (red line), the interquartile range (box), and the minimum and maximum (whiskers) after exclusion of outliers (open circles). For accuracy, positive scores indicate higher performance in the tDCS session. For RT, negative scores indicate faster performance in the tDCS session. For RT variance, negative scores indicate less variance in the tDCS session.

**Figure 4** summarizes our findings, showing that overall subjects performed better in the tDCS session (increases proportion correct, decreases reaction times, and decreases variance of the reaction times) than in the baseline session, irrespective of the type of stimulation (anodal or sham).

### Bayesian Analysis

The lack of significant interaction effects obtained in the traditional null hypothesis testing, as presented above, only suggests absence of evidence for an effect of tDCS on implicit categorization learning, but, importantly, no actual evidence of absence (Altman and Bland, 1995). The Bayesian analysis, however, did provide evidence against such interaction effects (see **Table 1**). We observed that the main model (including only the main effect of stimulation) is more plausible than the extended model (including the interaction between block and stimulation) for both response times and accuracy, as indicated by Bayes factors larger than 1 (6.36 and 2.26, respectively).

**Table 1:** Bayesian results, showing the Akaike Information Criterion (AIC) for each of the three models; the Bayes Factor (BF) comparing the null model vs. the main model (BF > 1 means the null model is more plausible), and the Bayes Factor comparing the main model vs. the extended model (BF > 1 means the main model is more plausible).

Model	Response time		Accuracy	
	AIC	Bayes factor	AIC	Bayes factor
Null model	-344.65		-938.59	
Main model	-344.96	0.73	-937.12	4.37
Extended model (including interaction)	-343.11	6.36	-936.30	2.26

## Discussion

In this study, we investigated the effects of anodal cerebellar tDCS in an implicit categorization task. Based on the reported effects of anodal tDCS (Jacobson et al., 2012) and results of our pilot study (Verhage et al., 2014), we hypothesized that anodal cerebellar tDCS will enhance learning during an implicit categorization task. Compared to the baseline session, subjects improved on their overall performance in the second (tDCS) session, showing increased accuracy scores, reduced reaction times, and reaction time variance, but this was independent of the type of stimulation (sham or anodal). As for learning, we did observe a small interaction effect, but *post hoc* comparisons failed to show any significant differences between anodal and sham stimulation over blocks. Additional Bayesian analysis provided evidence against an effect of anodal tDCS on learning over blocks. We, therefore, conclude that anodal cerebellar tDCS does not modulate performance and learning in an implicit categorization learning task.

The lack of tDCS effects on categorization performance is not that surprizing as its effects on cognition are still debated even for cortical tDCS (Horvath et al., 2015). Recently, we also reported such absences of effect of cerebellar tDCS stimulation on the N-back memory task (van Wessel et al., 2016) and probabilistic categoraztion learning (Seyed Majidi et al., 2017). This lack of a cerebellar tDCS effect on cognitive tasks could indicate that the cerebellum is not involved in cognition in general. This is, however, unlikely since several imaging studies (Nitsche et al., 2003; Blackwood et al., 2004; Hayter et al., 2007; Tomasi et al., 2007; Helie et al., 2010; Stoodley et al., 2012; Balsters et al., 2013; Lam et al., 2013; Davis et al., 2014; E et al., 2014) have shown cerebellar activity during cognitive tasks, including the task of categorization learning (Patalano et al., 2001; Milton et al., 2009). Anatomical evidence also supports the idea that the cerebellum is involved in fine-tuning processes in the prefrontal cortex (Kelly and Strick, 2003; Balsters et al., 2010). Moreover, anatomical connections between the cerebellum and the prefrontal cortex are likely to be cognitive in nature (Ramnani, 2006; Ito, 2008). Another explanation for not finding an effect of stimulation is that cerebellar tDCS is unable to modulate cognitive functions. However, this does not explain the positive effects found on various other cognitive tasks in earlier cerebellar tDCS research (Ferrucci et al., 2008; Pope and Miall, 2012; Boehringer et al., 2013). Furthermore, the lack of tDCS effects in our study are somewhat surprising given the reported effects of cerebellar tDCS in several motor tasks in which the cerebellum plays an important role. It is reported that anodal tDCS on the cerebellum enhances, for instance, learning of hand movement control in visuo-motor adaptation (Galea et al., 2011) and force-field adaptation tasks (Herzfeld et al., 2014). Cerebellar tDCS also seems to affect locomotor adaption (Jayaram et al., 2012). Therefore, one could have expected that cerebellar tDCS has similar effects on cognitive learning given the uniform architecture and the overall capability to process both motor and cognitive information (Kawato, 1999; Ramnani, 2006; Ito, 2008).

In our view, the most likely explanation for lack of cerebellar tDCS effect obtained here is that the cerebellum is not that critically involved in this type of cognitive learning. It is assumed that implicit learning in information-integration tasks is dominated by an implicit procedural-learning-based system, which in turn is mediated by the caudate nucleus (Ashby et al., 1998; Ashby and Ell, 2001) and the role of the cerebellum might be less prominent. Therefore, cerebellar tDCS could be less likely to modulate performance on our task. So, although it is widely acknowledged that the cerebellum is involved in cognition (Koziol et al., 2014), it remains to be elucidated how the cerebellum contributes to specific cognitive processes.

A general problem in tDCS research is the lack of standardized tDCS protocols. Furthermore, whether there is an effect of tDCS could depend on the level of task complexity (simple motor behavior vs. complex cognitive reasoning), stimulation intensity, and/or the side of the stimulation. This could account for the conflicting outcomes reported in the tDCS literature. Yet, this does not explain the conflicting results of our pilot study, in which we observed an effect, and the current study, since identical tDCS protocols were used. This suggests that the positive effect of cerebellar tDCS found in the pilot study was observed by chance. Once more, this shows the importance of replication studies (Vannorsdall et al., 2016). A limitation of the current study is the use of a between-subject design, which is not ideal for tDCS research because of high between-subject variability (Li et al., 2015). Therefore, adopting a within-subjects design, where the test-retest effect is kept to a minimum, would be better for most tDCS studies. However, when it comes to study the effect of tDCS on categorization learning, a within-subject design is less feasible due to the inevitable changes in performance over sessions, irrespective of stimulation condition. In addition, randomizing the order of sham (or no) stimulation and anodal stimulation, might lead to problems with the assumed prolonged effects of tDCS when some subjects start with real stimulation. For instance, a single session of 13 min anodal tDCS could enhance excitability up to 60 min after DC stimulation (Nitsche and Paulus, 2001; Monte-Silva et al., 2013).

Future research should focus on developing robust tDCS protocols. An earlier study investigating the effect of tDCS over the motor cortex on corticospinal excitability showed large variability in subject’s responsiveness to tDCS, which is in line with similar non-invasive brain-stimulation studies. These results highlight the importance of robust tDCS protocols and the need to ascertain individual factors that determine tDCS responsiveness (Wiethoff et al., 2014). Furthermore, future research should first aim to replicate promising effects of tDCS or related brain-stimulation techniques [such as transcranial magnetic stimulation (TMS) or theta burst stimulation (TBS); see Picazio et al., 2013] on cognitive processes before investigating new ground.

## Conclusion

Anodal tDCS applied over the cerebellum does not facilitate performance on an implicit categorization task and suggest that the cerebellum does not play a substantial role in implicit categorization based on the integration of information. Since, we failed to replicate the positive results of our underpowered pilot study (Verhage et al., 2014), the present outcome also highlights the importance of replication with sufficient power.

## Ethics Statement

The study took place at the Department of Neuroscience at the Erasmus MC in Rotterdam. This study was carried out in accordance with the recommendations of the Erasmus MC with written informed consent from all subjects. All subjects gave written informed consent in accordance with the Declaration of Helsinki. The protocol was approved by the METC of the Erasmus MC.

## Author Contributions

MV, EA, OD, and JvdG were involved in conception and design; MV was involved in acquisition; MV, OD, and JvdG were involved in analysis; MV, OD, MF, and JvdG were involved in interpretation; MV, EA, and OD were involved in drafting; and MV, MF, and JvdG were involved in revision. All authors contributed to the final approval and accountability.

## Conflict of Interest Statement

The authors declare that the research was conducted in the absence of any commercial or financial relationships that could be construed as a potential conflict of interest.
